# Neoadjuvant HER2 inhibition induces *ESR1* DNA methylation alterations resulting in clinically relevant ER expression changes in breast cancers

**DOI:** 10.1002/cac2.12640

**Published:** 2024-12-15

**Authors:** Gavin P. Dowling, Gordon R. Daly, Aisling Hegarty, Michael Flanagan, Mihaela Ola, Ramón Fallon, Sinéad Cocchiglia, Vikrant Singh, Katherine M. Sheehan, Fiona Bane, Jason McGrath, Louise Watson, Sandra Hembrecht, Bryan Hennessy, Patrick G. Morris, Arnold D. K. Hill, Damir Varešlija, Leonie S. Young

**Affiliations:** ^1^ Department of Surgery RCSI University of Medicine and Health Sciences Dublin Ireland; ^2^ Department of Surgery Beaumont Hospital Dublin Ireland; ^3^ Beaumont RCSI Cancer Centre Beaumont Hospital Dublin Ireland; ^4^ Department of Pathology RCSI University of Medicine and Health Sciences Dublin Ireland; ^5^ School of Pharmacy and Biomolecular Sciences RCSI University of Medicine and Health Sciences Dublin Ireland

**Abbreviations**
β: beta value
ASCO/CAP: American Society of Clinical Oncology/College of American Pathologists
Bx: pre‐treatment breast tumors
CIs: confidence intervals
DFS: disease‐free survival
EMT: epithelial to mesenchymal transition
ER: estrogen receptor
FFPE: formalin‐fixed paraffin‐embedded
FISH: fluorescence in‐situ hybridization
HER2: human epidermal growth receptor‐2
HR: hazard ratios
IHC: immunohistochemistry
m: methylation intensity value
Mx: subsequent metastasis
OS: overall‐survival
pCR: pathologic complete response
PR: progesterone receptor
Rx: metastatic surgical resection
Sx: post‐treatment resection specimen

1

The expression of estrogen receptor (ER) and human epidermal growth factor receptor‐2 (HER2) in breast cancer can change in response to treatment and pivotally influence tumor behavior and clinical management [1]. Receptor discordance has been observed at various distant metastatic site (bone, lung, liver, and brain), with a routine loss of ER and gains in HER2 reported [2]. This receptor discordance can influence tumor responsiveness to both HER2 inhibitor and endocrine therapies. Although the mechanisms underlying receptor expression changes are not fully understood, we recently reported gains in *ESR1* promoter hypermethylation as a potential driver of ER loss during disease progression [3]. In this study, mechanisms underlying altered receptor expression and associated disease outcomes were examined following neoadjuvant trastuzumab treatment.

We investigated the impact of HER2 inhibition on ER expression. From a cohort of 2,917 patients, 527 tumors were HER2‐positive. Of these, 161 patients received neoadjuvant trastuzumab with systemic chemotherapy (Supplementary Figure S1, Supplementary Table S1). In this cohort (*n* = 161), 89 patients (55.3%) achieved pathological complete response (pCR), while 72 patients (44.7%) had residual disease, and 18 patients developed metastases (Clinical Cohort, Figure 1A, Supplementary Table S1). A statistically significant higher proportion of patients with ER‐negative tumors achieved pCR compared to their ER‐positive counterparts (*P* = 0.016, Supplementary Table S1), consistent with previous studies [4]. In patients with residual disease, the most notable finding was the observed gain of ER protein expression and loss of HER2 in a number of patients (33% and 17%, respectively; Figure 1B). ER protein status remained unchanged in 38 patients (53%), with a loss of ER observed in 8 patients (11%) (Figure 1B).

**FIGURE 1:**
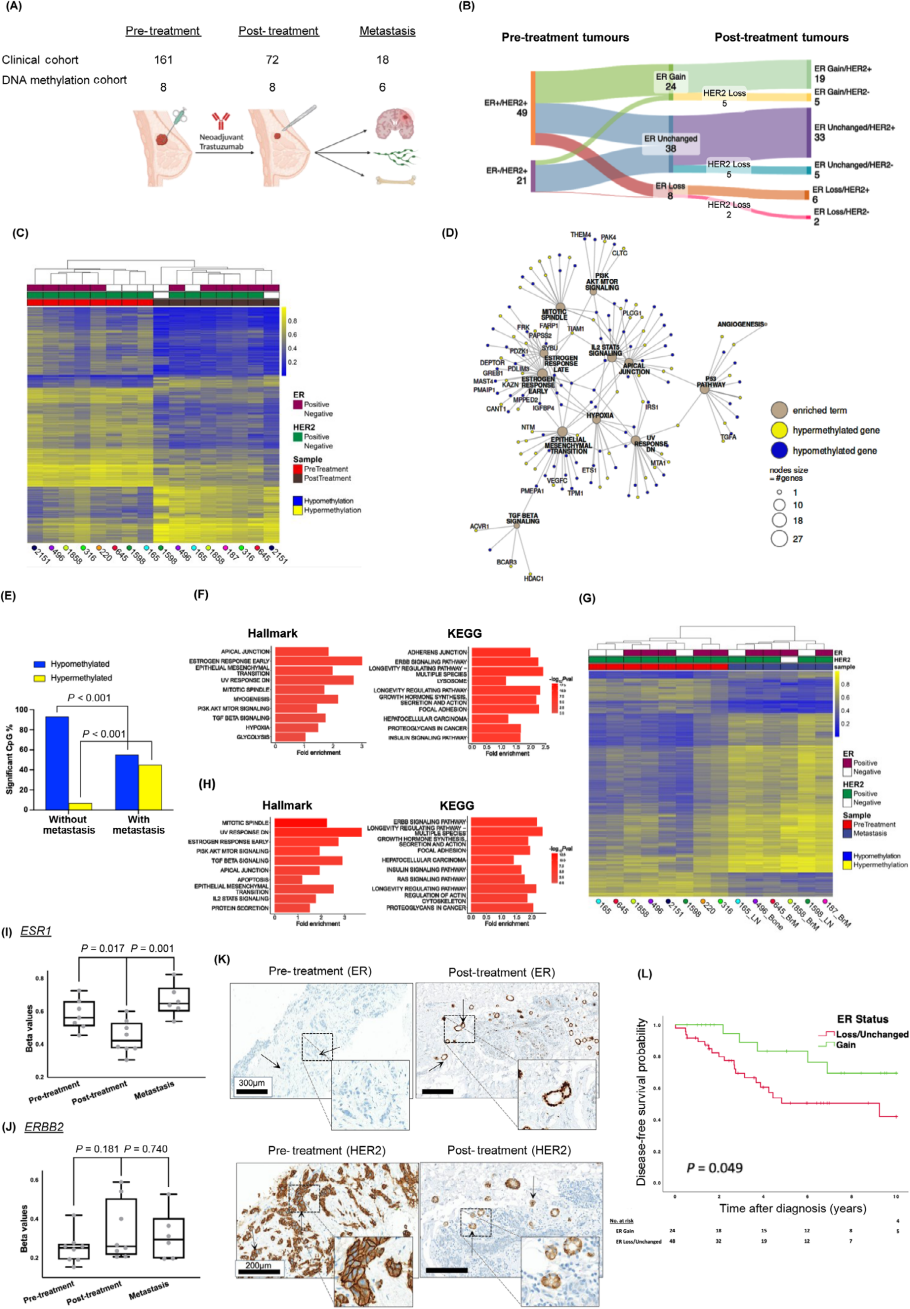
HER2 inhibition induces *ESR1* DNA methylation alterations. (A) A table summarizes the number of patients in the clinical study: pre‐treatment (*n* = 161), post‐treatment (*n* = 72), and metastasis (*n* = 18), along with the number of samples that underwent EPIC DNA methylation analysis: pre‐treatment (*n* = 8), post‐treatment (*n* = 8), and metastasis (*n* = 6). Metastatic samples included brain (*n* = 3), bone (*n* = 1), and axilla (*n* = 2). Of the pre‐treatment samples, 7 patients have matching post‐treatment sample. Among the metastatic samples, 5 have matching pre‐treatment and post‐treatment samples, while 1 has a matching post‐treatment sample only. The schematic illustrates the neoadjuvant therapeutic treatment and surgical intervention, created with https://www.biorender.com/. (B) A Sankey plot of ER and HER2 protein expression level changes in matched pre‐treatment and post‐treatment tumor samples visualizes dynamic changes in receptor expression following neoadjuvant trastuzumab. Created with https://sankeymatic.com/. (C) An unsupervised hierarchical clustering heatmap of differentially methylated genes (2,854 total) is shown. Methylation status is represented by a color scale, where dark blue indicates hypomethylation (835 CpG sites) and yellow indicates hypermethylation (2,019 CpG sites). Pre‐treatment samples (left) are represented in red, and post‐treatment (right) are represented in brown. Increased global hypomethylation is observed in post‐treatment samples. The x‐axis is annotated with patient identifiers and corresponding sample type. Bx, pre‐treatment biopsy; Sx, post‐treatment surgical resection. Paired samples are indicated by matching‐colored dots. (D) Network interaction graph of the top 12 Hallmark pathways of DMGs in post‐treatment samples compared to pre‐treatment samples. (E) Bar plot showing the distribution of significant CpG probes in post‐treatment methylation profile (blue: hypomethylated; yellow: hypermethylated) in samples with and without subsequent metastasis (*P* < 0.001, Fisher's exact test). (F) Hallmark and Kyoto Encyclopedia of Genes and Genomes (KEGG) pathway analysis of DMGs in post‐treatment versus pre‐treatment patients. The top 10 statistically significant pathways (*P* < 0.05) are shown. (G) Unsupervised hierarchical clustering heatmap of DMGs in pre‐treatment and metastatic samples. Methylation status is indicated by a color scale, where dark blue indicates hypomethylation (5,014 CpG sites), and yellow indicates hypermethylation (9,118 CpG sites). The x‐axis is annotated with individual patient identifiers and corresponding sample types. Bx, pre‐treatment biopsy; Rx, post‐treatment surgical resection of metastasis. Paired samples are indicated with matching‐colored dots. Heatmap shows significantly increased hypermethylation in metastatic samples compared to pre‐treatment samples. (H) Hallmark and KEGG pathway analysis of DMGs between pre‐treatment and post‐treatment in patients who subsequently developed metastatic disease. The top 10 statistically significant pathways (*P* < 0.05) are shown. (I) Boxplots for the *ESR1*‐cg01715172 EPIC probe show significant decreases in methylation levels post‐treatment (*P* = 0.0169) and subsequent increases in metastasis (*P* = 0.0014). (J) Boxplots for the *ERBB2*‐cg15227682 EPIC probe show no significant methylation changes post‐treatment (*P* = 0.181) or in metastasis (*P* = 0.7402). (I‐J) Data points represent individual samples, and boxes indicate the median and interquartile ranges. (K) Immunohistochemistry (IHC) staining of ER (top) and HER2 (bottom) in matched pre‐treatment and post‐treatment tumor tissues. Tumor areas are annotated with arrows. Black scale bars show 300µm and 200µm, respectively. (L) Kaplan‐Meier plot of disease‐free survival (DFS) for patients who gained ER positivity in post‐treatment residual disease compared to pre‐treatment biopsies (Gain HR = 0.37, 95% CI = 0.14‐0.99, *P* < 0.05). Abbreviations: Bx, pre‐treatment biopsy; DMG, differentially methylated gene; ER, estrogen receptor; HER2, human epidermal growth receptor 2; IHC, immunohistochemistry; KEGG, Kyoto Encyclopedia of Genes and Genomes; Rx, post‐treatment surgical resection of metastasis; Sx, post‐treatment surgical resection.

Epigenetic modifications are a potential mechanism underlying this observed receptor discordance. In this study, the role of DNA methylation in altered receptor expression was investigated (DNA methylation cohort, Figure 1A). Global DNA methylation was assessed in pre‐ and post‐treatment samples (*n* = 16; Supplementary Table S2), of which 7 patient tumors were matched (biopsy and resection). Differentially methylated gene (DMG) analysis segregated patient tumors into pre‐ or post‐treatment groups, illustrating the dominance of hypomethylation in post‐treatment patient tumors (Figure 1C). Notably, pathway analysis identified *Estrogen Response Early*, *Epithelial Mesenchymal Transition* (*EMT*), *ERRB*, *AMPK*, and *RAS* signaling (*P* < 0.05) as statistically significant pathways (Supplementary Figure S2A‐B). Network graph analysis revealed *Estrogen Response Early*, *Estrogen Response Late* and *EMT* as hub pathways (Figure 1D). Estrogen‐responsive genes such as *GREB1, FRK, IGFBP4* and *IRS1* were predominantly hypomethylated, while oncogenic genes such as *VEGFC, DEPTOR, TIAM1*, and *HDAC1* were hypermethylated (Figure 1D, Supplementary Table S3).

Analysis of pre‐ and post‐neoadjuvant treated tumors from patients with no metastases (good outcomes) compared to those with metastases (poor outcomes) revealed significant divergent differential methylation profiles (*P* < 0.001; Figure 1E, Supplementary Table S4). In patients with subsequent metastases, reduced hypomethylation and enhanced hypermethylation were observed (Figure 1E). Pathway analysis of DMGs highlighted *PI3K/AKT/mTOR* (Hallmark) and *ERRB2* (KEGG) as statistically significant pathways (*P* < 0.05; Figure 1F). Network graph analysis of hypermethylated Hallmark pathways (top 12) identified *PI3K/AKT/mTOR* signaling as a hub pathway with poor outcomes (Supplementary Figure S2C, Supplementary Table S4). Notably, differential hypermethylation was detected in genes such as *SMAD2, AKT1, PAK4, EGFR, BRCA2, NF1* and *PIK3R3* (Supplementary Figure S2C).

We also characterized DNA methylation profiles in pre‐treatment biopsies and metastatic tumors. Global gains in hypermethylation were observed upon metastasis in both ER‐positive and ER‐negative matched tumors (Figure 1G). Pathway analysis revealed alterations in *PI3K/AKT/MTOR* (Hallmark), *ERBB*, *focal adhesion*, and *Ras* (KEGG) signaling pathways (*P* < 0.05; Figure 1H, Supplementary Table S5). Network analysis of DMGs identified *PI3K/AKT/mTOR* signaling as a hub pathway, with hypermethylation of *SMAD2, MAPK8, PAK4*, and hypomethylation of *PRKCA, EGFR*, and *PIK3CD* (Supplementary Figure S2D, Supplementary Table S6). Of note, global hypermethylation was more pronounced in metastatic tumors compared to post‐treatment tumors (Supplementary Figure S3A), with differential methylation affecting key oncogenic pathways including *EMT*, *PI3K/AKT/MTOR*, and *estrogen response* (Supplementary Figure S3B).

Consistent with global hypomethylation observed post‐neoadjuvant treatment (Figure 1C), *ESR1* probe cg01715172 was identified as a statistically significant hypomethylated promoter CpG in *ESR1* post‐treatment (*P* = 0.0169), with subsequent significant gains in *ESR1* promoter hypermethylation observed upon metastasis (*P* = 0.0014), (Figure 1I). These changes reflect altered CpG DNA methylation across *ESR1* (Supplementary Figure S4). Conversely, *ERBB2* promoter hypermethylation at cg15227682 was identified post‐treatment, with promoter hypomethylation observed upon metastasis, though these differences did not reach statistical significance (Figure 1J). At a functional level, alterations in the promoter methylation status of *ESR1* and *ERBB2* were validated at the protein level, with relative gains in ER expression and a loss of HER2 expression observed post‐neoadjuvant treatment (Figure 1K, Supplementary Table S7). Trastuzumab‐induced *ESR1* hypomethylation and related ER expression gains were further validated in HER2‐positive cell line models, SKBR3 and T347 cells. No alterations were observed in HER2 non‐amplified/trastuzumab‐insensitive LY2 cells (Supplementary Figure S5).

The clinical relevance of protein expression changes as a consequence of dynamic methylation status of key breast cancer receptors ER and HER2 was determined. In patients with residual disease, gains in ER protein positivity following neoadjuvant therapy were associated with enhanced disease‐free survival (DFS) (Gain HR = 0.37, 95% CI = 0.14‐0.99, *P* < 0.05) (Figure 1L) and showed a trend toward an association with overall survival (OS) (Gain HR = 0.40, 95% CI = 0.15‐1.10, *P* < 0.07, Supplementary Figure S6A). However, alterations in HER2 status post‐neoadjuvant treatment did not have a statistically significant impact on either DFS or OS in this patient population (Supplementary Figure S6B‐C).

We demonstrate dynamic alterations in receptor status in response to trastuzumab. Although the number of patient samples in this study is relatively limited, the data reported here support DNA methylation as a driver of expression changes, with global hypomethylation following neoadjuvant treatment and hypermethylation upon metastasis. While differential methylation of key signaling pathways is conserved between post‐treatment primary surgery and metastasis, the direction of methylation shifts in core pathway genes, including *ESR1*, as well as known tumor suppressor genes, such as *FRK*, *RARB*, and *SSBP2* [5], from hypomethylation post‐neoadjuvant treatment to hypermethylation upon metastasis, ultimately driving an aggressive clinical phenotype.

The observed epigenetic changes in *ESR1* and *ERBB2* highlight the modifying nature of methylation in response to treatment and disease progression. Post‐treatment hypomethylation of *ESR1* may contribute to maintaining sensitivity to endocrine therapy, whereas hypermethylation during metastatic stages suggests a shift towards a more resistant phenotype.

Preclinical and clinical findings have demonstrated that, for HER2/ER‐positive tumors, treatment with HER2‐directed therapy in isolation increases ER expression through crosstalk between these receptors [6]. While this upregulation of ER and ER‐related genes leads to a compensatory ‘escape’ pathway, it simultaneously creates an additional therapeutic target, with evidence that sustained anti‐HER2 therapy sensitizes tumor cells to endocrine therapies [7]. Therefore, it has been suggested that dual blockade of HER2 and ER pathways may be necessary in HER2/ER‐positive tumors to sustain an antitumor effect [7, 8]. Concomitant treatment with HER2‐directed therapy and endocrine therapy has been reported to have greater antitumor activity than HER2‐targeted therapy alone in multiple preclinical models [9]. Clinical trial data also suggest that the combination of endocrine therapy and anti‐HER2 therapy is an effective therapeutic strategy in this cohort [10]. Therefore, repeat receptor analysis after treatment is crucial, as patients switching from ER‐negative to ER‐positive can benefit from the addition of endocrine therapy to their treatment regimen.

We report that an increase in ER expression post‐neoadjuvant therapy is statistically significantly associated with better survival outcomes compared to tumors with decreased or unchanged ER expression. This suggests that the interplay between ER and HER2 is highly dynamic, with its influence on tumor behavior being more rapid in response to perturbations in cellular signaling than is currently understood. Global DNA methylation patterns shift in response to treatment and subsequent metastasis, altering the tumor phenotype. Consequential changes in core ER signaling not only influences outcomes but also provide opportunities for meaningful clinical intervention. Unlocking DNA methylation as a key process in breast cancer progression can provide important insights into the consequences of treatment and aid in the development of new therapeutic strategies.

### AUTHOR CONTRIBUTIONS

Damir Varešlija and Leonie S. Young: study concept and design. Gavin P. Dowling, Gordon R. Daly, Damir Varešlija, Aisling Hegarty, Sinéad Cocchiglia, Sandra Hembrecht, Michael Flanagan, Mihaela Ola, Ramón Fallon, Fiona Bane, Vikrant Singh, Katherine M. Sheehan, Louise Watson, Jason McGrath, and Leonie S. Young: acquisition, analysis, or interpretation of data. Aisling Hegarty, Sinéad Cocchiglia, Patrick G. Morris, Bryan Hennessey, and Arnold D.K Hill: provision of administrative, technical, or material support. Gavin P. Dowling, Gordon R. Daly, Sinéad Cocchiglia, Damir Varešlija, and Leonie S. Young: drafting of the manuscript. All authors: critical revision of manuscript.

## CONFLICT OF INTEREST STATEMENT

The authors declare no conflict of interest.

## FUNDING INFORMATION

We kindly acknowledge the funding support from Science Foundation Ireland Frontiers for the Future Award (19/FFP/6443), Science Foundation Ireland Strategic Partnership Program, Precision Oncology Ireland (18/SPP/3522) (L.S.Y.), Breast Cancer Now Fellowship Award with the generous support of Walk the Walk (2019AugSF1310) (D.V.), Science Foundation Ireland (20/FFP‐P/8597) (D.V.), Breast Cancer Ireland program Grant (18239A01) (L.S.Y.).

## ETHICS APPROVAL AND CONSENT TO PARTICIPATE

Written and informed consent was acquired prior to collection of patient tumor tissue under The Royal College of Surgeons Institutional Review Board approved protocol (CTI 09/07). All clinical material was collected as part of the prospective observational clinical trial NCT01840293 (https://clinicaltrials.gov).

## Supporting information



Supporting Information

Supporting Information

## Data Availability

Raw DNA methylation data files can be found at the Gene Expression Omnibus (GEO) website under accession number GSE283272.
