# Supplementary material for: Neoadjuvant HER2 inhibition induces ESR1 DNA methylation alterations resulting in clinically relevant ER expression changes in breast cancers
Source: Cancer Commun (Lond). 2024 Dec 15;45(2):198–202. doi: 10.1002/cac2.12640 (PMC11833670; doi:10.1002/cac2.12640)
Supplement: Supplementary file 1 — Supporting Information [file CAC2-45-198-s001.docx]

**Supplementary Materials**

**Neoadjuvant HER2 inhibition induces *ESR1* DNA methylation alterations resulting in clinically relevant ER expression changes in breast cancer**

Gavin P. Dowling^1,2,*^, Gordon R. Daly^1,2,*^, Aisling Hegarty^1,2,3^, Michael Flanagan^1,2^,

Mihaela Ola^1^, Ramón Fallon^1^, Sinéad Cocchiglia^1^, Vikrant Singh^1^, Katherine M. Sheehan^4^, Fiona Bane^1^, Jason McGrath^1^, Louise Watson^1^, Sandra Hembrecht^1,2^, Bryan Hennessy^3^, Patrick G. Morris^3^, Arnold D.K. Hill^1,2,3^, Damir Varešlija^3,5,#^, Leonie S. Young^1,3,#^

^1^Department of Surgery, RCSI University of Medicine and Health Sciences, Dublin, D02 YN77, Ireland

^2^Department of Surgery, Beaumont Hospital, Dublin, D09V2N0, Ireland

^3^ Beaumont RCSI Cancer Centre, Beaumont Hospital, Dublin, D09V2N0, Ireland

^4^ Department of Pathology, RCSI University of Medicine and Health Sciences, Dublin, D02 YN77, Ireland

^5^School of Pharmacy and Biomolecular Sciences, RCSI University of Medicine and Health Sciences, Dublin, D02 YN77, Ireland

^*^Gavin P. Dowling and Gordon R. Daly contributed equally to this work.

^#^Corresponding authors:

Leonie S. Young, Department of Surgery, RCSI University of Medicine and Health Sciences, Dublin, D02 YN77, Ireland. Email: [lyoung@rcsi.ie](mailto:lyoung@rcsi.ie)

Damir Varešlija, School of Pharmacy and Biomolecular Sciences, RCSI University of Medicine and Health Sciences, Dublin, D02 YN77, Ireland. Email: damirvareslija@rcsi.ie

**Supplementary Materials and Methods**

**Ethics**

Written and informed consent was obtained prior to the collection of patient tumor tissue under The Royal College of Surgeons Institutional Review Board approved protocol (CTI 09/07). All clinical material was collected as part of the prospective observational clinical trial NCT01840293 (https://clinicaltrials.gov).

**Study design**

The study population included female breast cancer patients diagnosed with HER2-positive breast cancer who received treatment with neoadjuvant trastuzumab, either alone or in combination with systemic chemotherapy. Clinicopathological and survival data were collected for all patients from a prospectively maintained database. Patients were excluded from the survival analysis if they were lost to follow-up (> 6 months) or died from another known cause. No patients in this study, with one exception, received adjuvant hormonal therapy.

**Histopathologic and immunohistochemistry appraisal**

Tumor specimens were assessed in accordance with the 2010 American Society of Clinical Oncology/College of American Pathologists (ASCO/CAP) histopathological consensus guidelines for ER and progesterone receptor (PR) status, where ≥1 % of tumor nuclei positive was reported as ER-positive [1]. HER2 status was determined using immunohistochemistry (IHC); patients with a score of 2+ on IHC proceeded for fluorescence in-situ hybridisation (FISH) to confirm HER2 status. The percentage positivity of ER staining for diagnostic pre-treatment core tumor biopsy and corresponding residual primary breast tumour was assessed by a single consultant histopathologist. Residual tumours were categorised as having a gain or loss of ER if the change in ER staining positivity compared to the pre-treatment biopsy was ≥ 10 %. Residual tumours were categorised as losing HER2 positivity if they had a score of 0, 1+ or 2+ with negative FISH on reassessment. A pCR was defined as the absence of residual invasive cancer of the completely resected breast specimen and all sampled regional lymph nodes following completion of neoadjuvant therapy (ypT0/ Tis ypN0) [2].

**Statistical Analysis**

Clinicopathological characteristics and treatment outcomes were analyzed using descriptive statistics. Chi-squared (χ2), Fisher’s exact (¶), and one-way analysis of variance (ANOVA, α) tests were used, as appropriate. All tests of significance were 2-tailed, with *P* < 0.05 indicating statistical significance. Kaplan-Meier survival curves and log-rank analyses were performed to determine the effect of ER staining positivity change and HER2 status change on survival. Cox-regression analysis was performed and expressed as hazard ratios (HR) with 95 % confidence intervals (CIs). Individual *ESR1* and *ERBB2* promoter CpG methylation probes were compared in pre-treatment vs. post-treatment and pre-treatment vs. metastasis using paired t-tests. All analyses were performed using the Statistical Package for Social Sciences (SPSS^TM^), version 29.0 (International Business Machines Corporation, Armonk, New York).

**DNA extraction**

DNA was extracted from formalin-fixed paraffin-embedded (FFPE) tissue from patient-matched, pre-treatment breast tumors (Bx), post-treatment resection specimen (Sx), and subsequent metastasis (Mx). This extraction was performed using the Qiagen QiAMP DNA FFPE kit and run on the QIAcube, following manufacturer’s standard protocols.

**DNA Methylation Profiling**

The Infinium Methylation EPIC array (Illumina, San Diego, CA, USA) was used with the Infinium MethylationEPIC v1.0 B5 manifest published by Illumina (<http://www.Illumina.com>) to measure the DNA methylation profile of 850,000 CpGs for each sample. All the microarray chips were scanned using the Illumina HiScan system. Array processing and Beadstudio analysis were conducted through Genuity Science Ltd (Ireland) and Beaumont Hospital (Dublin, Ireland).

Beta (β) and methylation intensity (m) values were extracted, normalized, and batch corrected to determine all samples' differential methylation probes (DMP) using the ChAMP package on the R platform [3].

**Quality Control, Batch correction and Differential Methylation Analysis**

Quality control was performed on the raw data (idat files); probes were filtered with a detection *P* value above 0.01 in all samples. Furthermore, probes with a bead count of less than 3 in at least 5% of the samples were removed. Also, probe sequences which aligned at multiple sites and potential probes with SNPs were excluded from the analysis using the ChAMP package [3-5]. The raw data were normalized using Beta-mixture quantile normalization (BMIQ), and batch correction was performed using the combat function from the SVA package within the ChAMP pipeline [6, 7].

Differential methylated probe analysis was performed using the champ. DMP function in the ChAMP package, which utilizes the limma package, where the threshold for the Benjamini-Hochberg adjusted *P* values threshold was set to 0.05 [8]. A CpG was considered differentially methylated if the methylation difference between conditions (the beta value difference) was at least 0.2. Adjusted *P* value < 0.05 was used as the cutoff value in the Circos plot implemented by the shinyCircos package (<https://github.com/YaoLab-Bioinfo/shinyCircos>). The smoothScatter function from the R graphics package was used to create the colorimetric density plots. Unsupervised hierarchical clustering was performed in the heatmap plots using R's pheatmap package (<https://www.rdocumentation.org/packages/pheatmap/versions/1.0.12>) with normalized beta values from the significant differentially methylated probes, where the distance between samples was defined using the maximum linkage method, and the complete agglomeration method was used for clustering. Statistically significant CpGs were visualized using the interactive plot function DMP.GUI in the ChAMP package [8].

**Pathway Analysis**

Differentially methylated probe lists from the analysis were used to perform pathway analysis using the active subnetwork search (active_snw_search function) and the enrichment_analyses functions of the pathfindR package [9]. Two pathway collections were used: the Kyoto Encyclopedia of Genes and Genomes (KEGG) and the Human MSigDB Hallmark genesets [10, 11]. Enrichment charts or dot plots were then generated to show the top 12 most representative terms in each case using the Bonferroni correction with a threshold of 0.05. Network graph analysis was conducted using the igraph and ggplot2 R packages, and the results were visualised in term-gene plots.

**Cell Lines and Culture**

SKBR3 cells were purchased from American Type Culture Collection (ATCC) and were cultured in Roswell Park Memorial Institute (RPMI) 1640 media (R0883, Sigma) with 10% Fetal Calf Serum (FCS) (F7524, Merck) and 4 mmol/L of L-glutamine (G7513, Merck). LY2 cells were received as a gift from Dr. Robert Clarke (Georgetown University, Georgetown, United States) and were cultured in phenol red-free MEM (PRF-MEM) (51200046, Fisher Scientific), supplemented with 10 % charcoal dextran (F6765, Merck) stripped FCS (CDS-FCS) and 10^-8^ mol/L of 4-hydroxytamoxifen (4-OHT) (H7904, Merck). The brain metastatic patient-derived T347 cells were cultured in human breast epithelial cell media (HBEC) (HyClone™ DMEM/F12 1:1, SH30023.01, Cytiva), supplemented with 10 mmol/L HEPES (H3537, Merck), 5 % FCS (Supplier), 1X Insulin-Transferrin-Selenium (41400-045, Fisher Scientific), 1X Antibiotic-Antimycotic solution (15240096, Fisher Scientific), and 0.5 µg/mL hydrocortisone (H0888, Merck) [3]. Cells were incubated in a humidified incubator with 5% CO_2_, at 37°C.

**Western Blot Analysis**

SKBR3, T347 and LY2 cells were treated with 10µg/ml of trastuzumab (A2007, Selleckchem) for 8 and 24 hours, along with a vehicle control. Total protein was extracted using lysis buffer (0.1 % IGEPAL® CA-630 (I8896, Merck), 0.5 % deoxycholic acid (D2510, Merck), 0.1 % SDS (L3771, Merck)) and freshly supplemented with protease and phosphatase inhibitors (78442, Fisher Scientific). Lysates were centrifuged at 13,000 RPM for 20 minutes at 4°C, and the supernatant was collected for analysis. Pierce BCA Protein Assay Kit (23227, Fisher Scientific) was used to quantify protein concentration, with 30-90 µg of protein per sample loaded onto gels. The proteins were separated on Bolt™ Bis-Tris Plus WedgeWell™ 4 – 12 % gels (#NW04122BOX, Invitrogen) and transferred onto nitrocellulose membranes (GE10600015, GE Healthcare). Membranes were probed for ER-alpha (C1355, Merck Millipore) at a 1:1000 dilution, followed by incubation with anti-rabbit IgG horseradish peroxidase (HRP)-conjugated secondary antibody (7076, Cell Signaling Technology) at a 1:2,000 dilution. Membranes were then probed for beta-actin (A1978-100UL, Sigma) used at a 1:5,000 dilution, followed by incubation with anti-mouse IgG HRP-conjugated secondary antibody (7076, Cell Signaling Technology) at a 1:2,000 dilution. Bands were detected using enhanced chemiluminescence (ECL) (32106, Fisher Scientific) substrate on the Amersham Imager 680 Imaging System (GE Healthcare). Densitometric analysis was performed using ImageJ software. The intensity of the ER-alpha band in the treatment groups was normalized to the intensity of the beta-actin band by dividing the band of interest by the beta-actin band and normalizing to the control sample. All experiments were performed in triplicate.

**Methylation Specific-PCR Analysis**

SKBR3 cells were treated with 10 µg/mL of trastuzumab (A2007, Selleckchem) for 8 hours and 24 hours, alongside a vehicle control. DNA was extracted using the QIAamp DNA Mini Kit (Qiagen), and 2 µg of DNA was processed through the EZ DNA Methylation-Lightning Kit (Zymo Research, Freiburg, Germany) for sodium bisulfite conversion following the manufacturer's instructions. Methylation-specific PCR (MSP) was then conducted to differentiate unmethylated cytosine from methylated cytosine, selectively amplifying either methylated or unmethylated DNA using specific primers [12]. The primer sequences used for the methylated primer was ‘F: 5'-AGTAGGTGTTTTATTATTTGGAGAACG-3'

R: 5'-CGACGAAAAAACTTAACTCTAAACG-3' (Sequence start-end, 1404-1685) and the unmethylated primer sequence was “F: 5'-GTAGGTGTTTTATTATTTGGAGAATGA-3'

R: 5'-CCAACAAAAAAACTTAACTCTAAACAC-3' (Sequence start-end, 1405-1686). All primers were synthesized by Sigma-Aldrich. PCR amplification was performed with an initial denaturation at 95°C for 5 minutes, followed by 35 cycles (95°C for 30 seconds, 55°C for 45 seconds, and 72°C for 45 seconds), with a final extension at 72°C for 10 minutes. The PCR products were visualized on a 2% agarose gel.

**References**

1. Allison KH, Hammond MEH, Dowsett M, McKernin SE, Carey LA, Fitzgibbons PL, et al. Estrogen and Progesterone Receptor Testing in Breast Cancer: ASCO/CAP Guideline Update. J Clin Oncol. 2020;38(12):1346-66.

2. Litton JK, Regan MM, Pusztai L, Rugo HS, Tolaney SM, Garrett-Mayer E, et al. Standardized Definitions for Efficacy End Points in Neoadjuvant Breast Cancer Clinical Trials: NeoSTEEP. J Clin Oncol. 2023;41(27):4433-42.

3. Tian Y, Morris TJ, Webster AP, Yang Z, Beck S, Feber A, et al. ChAMP: updated methylation analysis pipeline for Illumina BeadChips. Bioinformatics. 2017;33(24):3982-4.

4. Nordlund J, Bäcklin CL, Wahlberg P, Busche S, Berglund EC, Eloranta ML, et al. Genome-wide signatures of differential DNA methylation in pediatric acute lymphoblastic leukemia. Genome Biol. 2013;14(9):r105.

5. Zhou W, Laird PW, Shen H. Comprehensive characterization, annotation and innovative use of Infinium DNA methylation BeadChip probes. Nucleic Acids Res. 2017;45(4):e22.

6. Teschendorff AE, Marabita F, Lechner M, Bartlett T, Tegner J, Gomez-Cabrero D, et al. A beta-mixture quantile normalization method for correcting probe design bias in Illumina Infinium 450 k DNA methylation data. Bioinformatics. 2013;29(2):189-96.

7. Leek JT, Johnson WE, Parker HS, Jaffe AE, Storey JD. The sva package for removing batch effects and other unwanted variation in high-throughput experiments. Bioinformatics. 2012;28(6):882-3.

8. Smyth GK. Linear models and empirical bayes methods for assessing differential expression in microarray experiments. Stat Appl Genet Mol Biol. 2004;3:Article3.

9. Ulgen E, Ozisik O, Sezerman OU. pathfindR: An R Package for Comprehensive Identification of Enriched Pathways in Omics Data Through Active Subnetworks. Front Genet. 2019;10:858.

10. Kanehisa M, Goto S. KEGG: kyoto encyclopedia of genes and genomes. Nucleic Acids Res. 2000;28(1):27-30.

11. Liberzon A, Birger C, Thorvaldsdóttir H, Ghandi M, Mesirov JP, Tamayo P. The Molecular Signatures Database (MSigDB) hallmark gene set collection. Cell Syst. 2015;1(6):417-25.

12. Li LC, Dahiya R. MethPrimer: designing primers for methylation PCRs. Bioinformatics. 2002;18(11):1427-31.

**Supplementary Figures Legend**


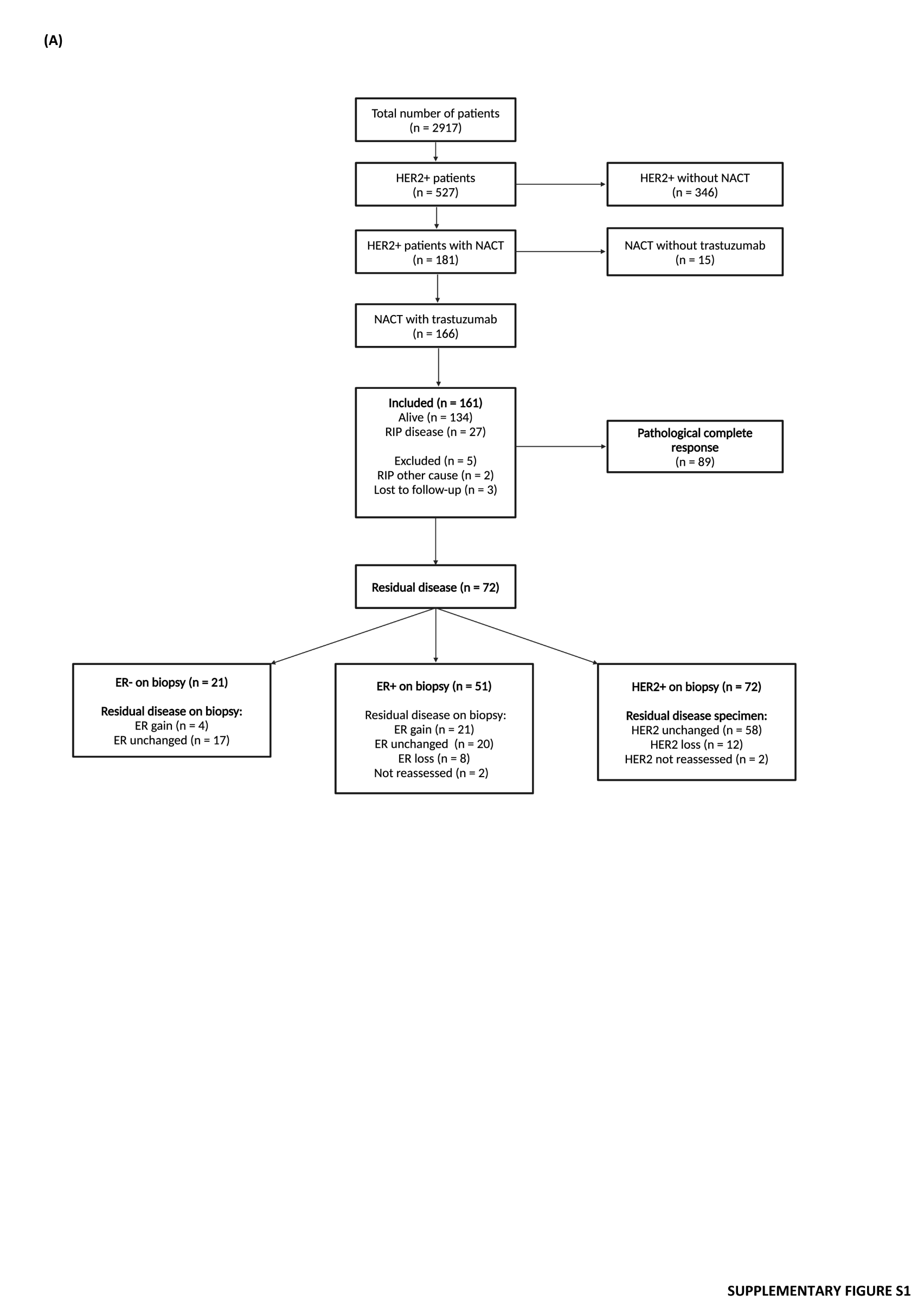


**Supplementary Figure S1. Patient selection for clinical study.**

Flow diagram summarizing patient selection for clinical study. From an initial cohort of 2,917 patients, 527 were HER2-positive (HER2^+^). Among these, 181 patients received neoadjuvant chemotherapy (NACT), and 166 also received neoadjuvant anti-HER2 therapy. After excluding 5 patients lost to follow-up, 161 patients were included in the analysis. Within this cohort, 89 patients achieved a pathological complete response (pCR) to NACT, while 72 had residual disease. Pre-treatment biopsies revealed that 21 patients were ER-negative (ER^-^) and 51 were ER-positive (ER^+^).

Abbreviations: HER2^+^, HER2-positive; NACT, neoadjuvant chemotherapy; pCR, pathological complete response; ER^-^, ER-negative; ER^+^, ER-positive.


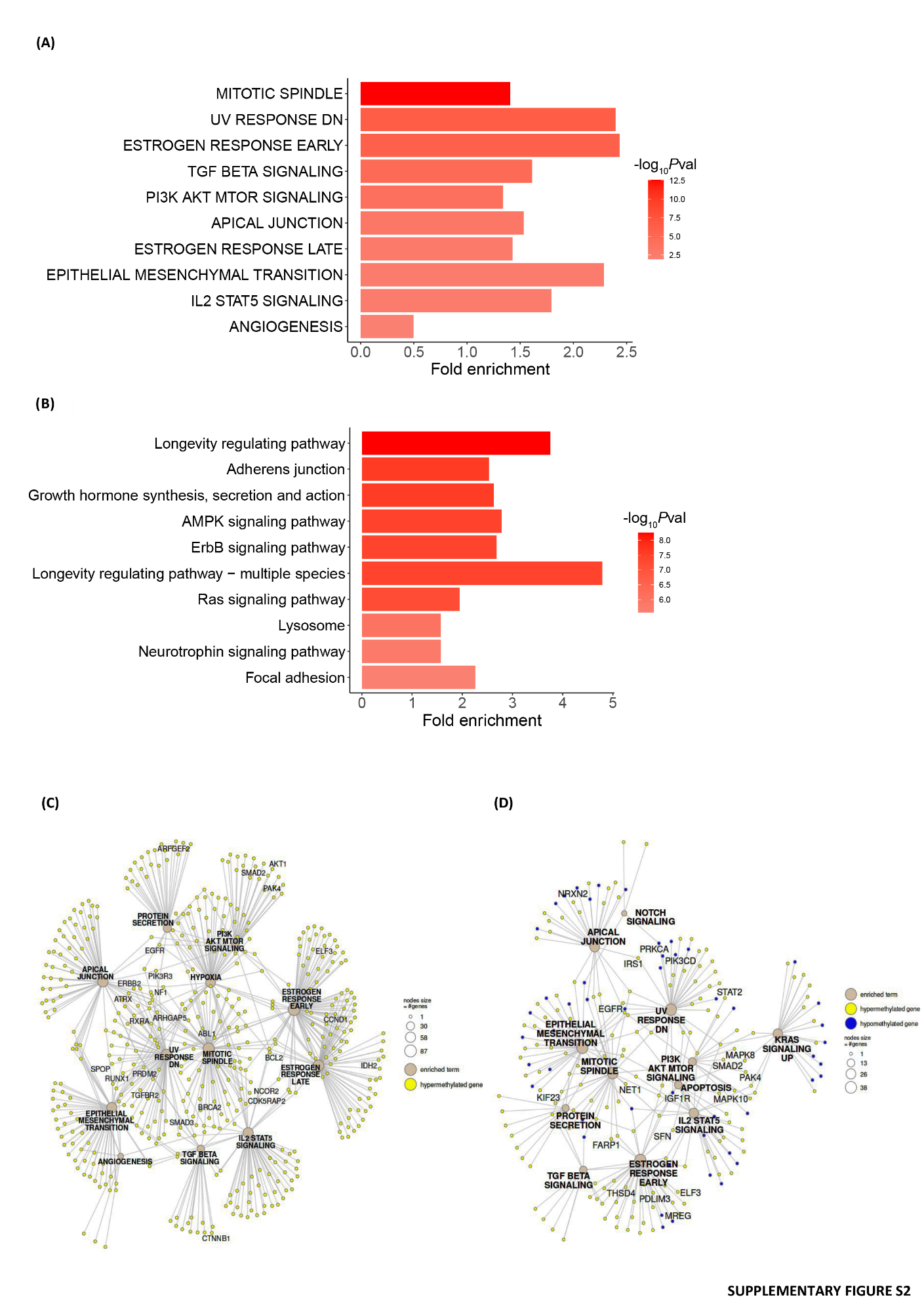


**Supplementary Figure S2. DNA methylation analysis.**

**(A)** Swimmer plot representation of top 10 Hallmark pathway analysis of differentially methylated genes in post-treatment versus pre-treatment samples.

**(B)** Swimmer plot representation of top 10 Kyoto Encyclopedia of Genes and Genomes (KEGG) pathway analysis of differentially methylated genes in post-treatment versus pre-treatment samples.

**(C)** Term-gene interaction graph of top 12 Hallmark pathways of differentially hypermethylated genes in patients with subsequent metastasis compared to pre-treatment samples. Increased kinase activity and reduced ER expression pathways are observed compared to Figure 1D.

**(D)** Term-gene interaction network of top 12 Hallmark pathways of differentially methylated genes in metastatic samples compared to pre-treatment samples.

Abbreviations: KEGG, Kyoto Encyclopedia of Genes and Genomes.


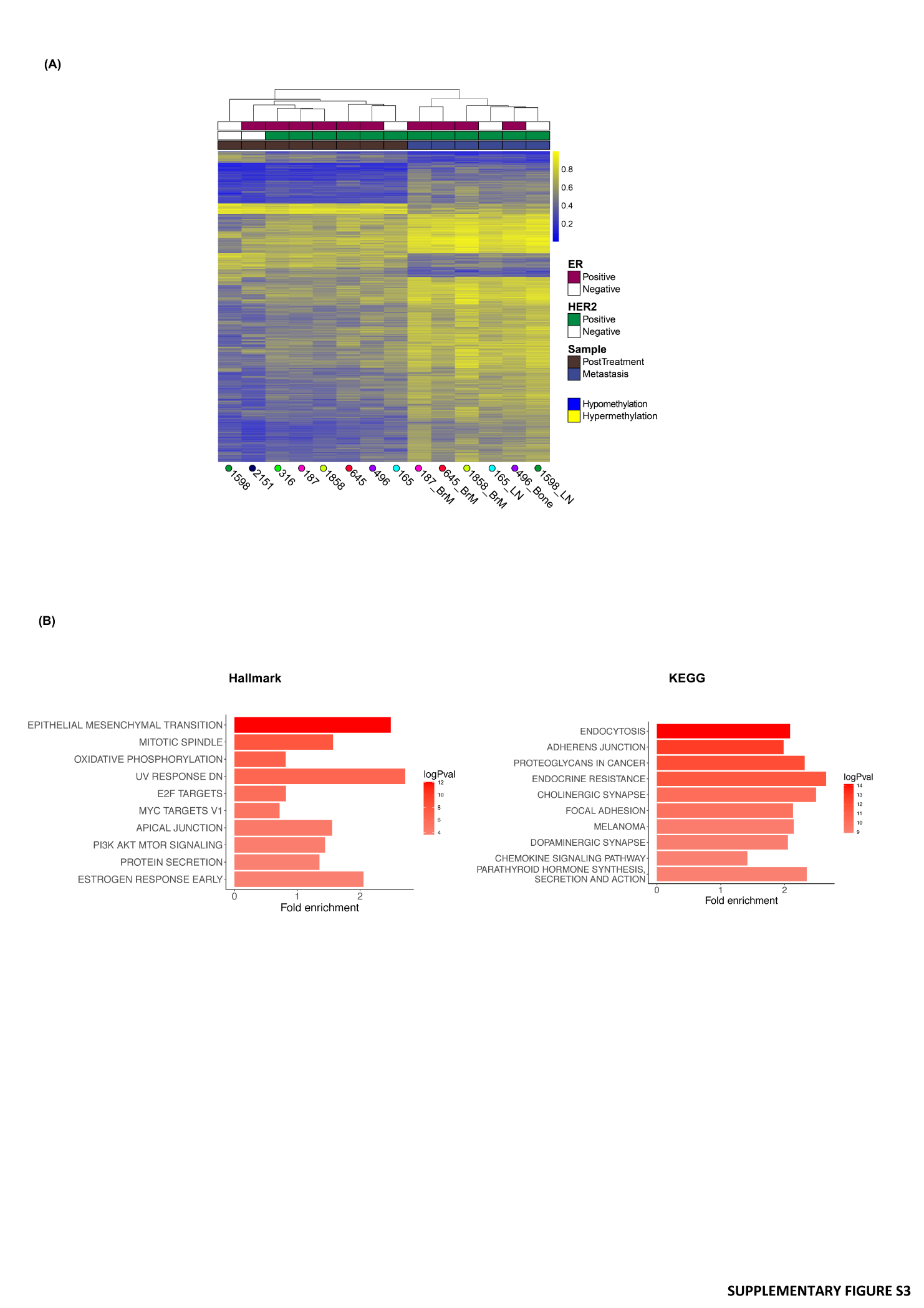


**Supplementary Figure S3. DNA methylation analysis of metastatic versus pre-treatment samples.**

1. Unsupervised hierarchical clustering heatmap of differentially methylated genes. Methylation status is indicated by a color scale where dark blue indicates hypomethylation and yellow indicates hypermethylation. Post-treatment (left) samples are represented by brown and metastasis (right) samples are represented by blue. Increased global hypermethylation is observed in metastatic samples. The x-axis is annotated with patient identification.
2. Hallmark and Kyoto Encyclopedia of Genes and Genomes (KEGG) pathway analysis of DMGs in metastatic versus post-treatment patients. The top 10 statistically significant pathways are shown (*P* < 0.05).

Abbreviations: KEGG, Kyoto Encyclopedia of Genes and Genomes.


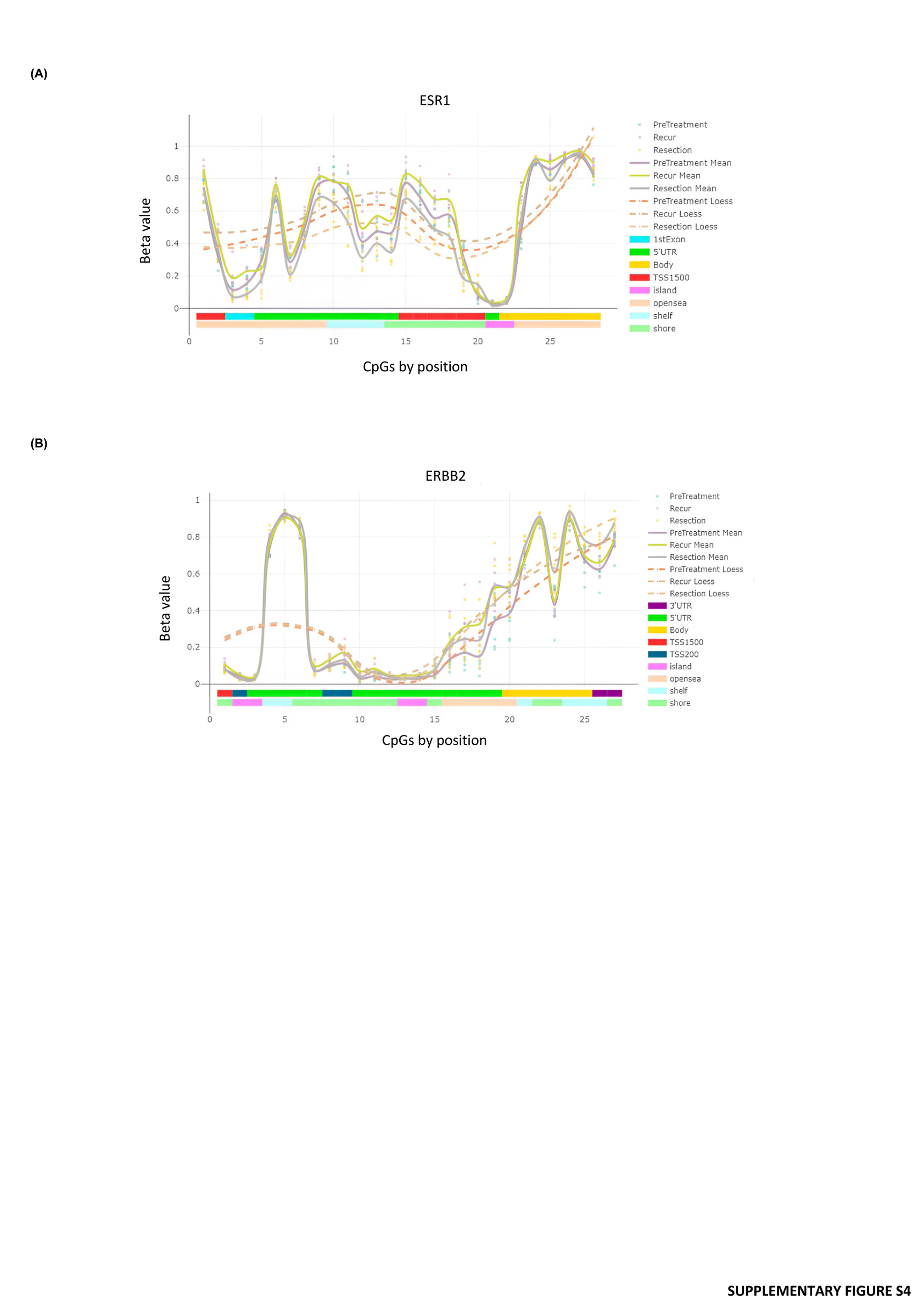


**Supplementary Figure S4. Statistically significant CpGs involved in (A) ESR1 and (B) ERBB2.**

1. Statistically significant CpGs involved in ESR1: The y-axis displays methylation beta values and the x-axis shows statistically significant CpGs sorted by their position on the chromosome. The distance between CpGs is equal. The individual beta values for each patient are represented by dots and color-coded by category (pre-treatment, post-treatment, metastases). The mean beta values and Loess curves for each category (pre-treatment, post-treatment, metastases) are also color-coded.
2. Statistically significant CpGs involved in ERBB2. The y-axis displays methylation beta values, and the x-axis shows statistically significant CpGs sorted by their position on the chromosome. The distance between CpGs is equal. The individual beta values for each patient are represented by dots and color-coded by category (pre-treatment, post-treatment, metastases). The mean beta values and Loess curves for each category (pre-treatment, post-treatment, metastases) are also color-coded.


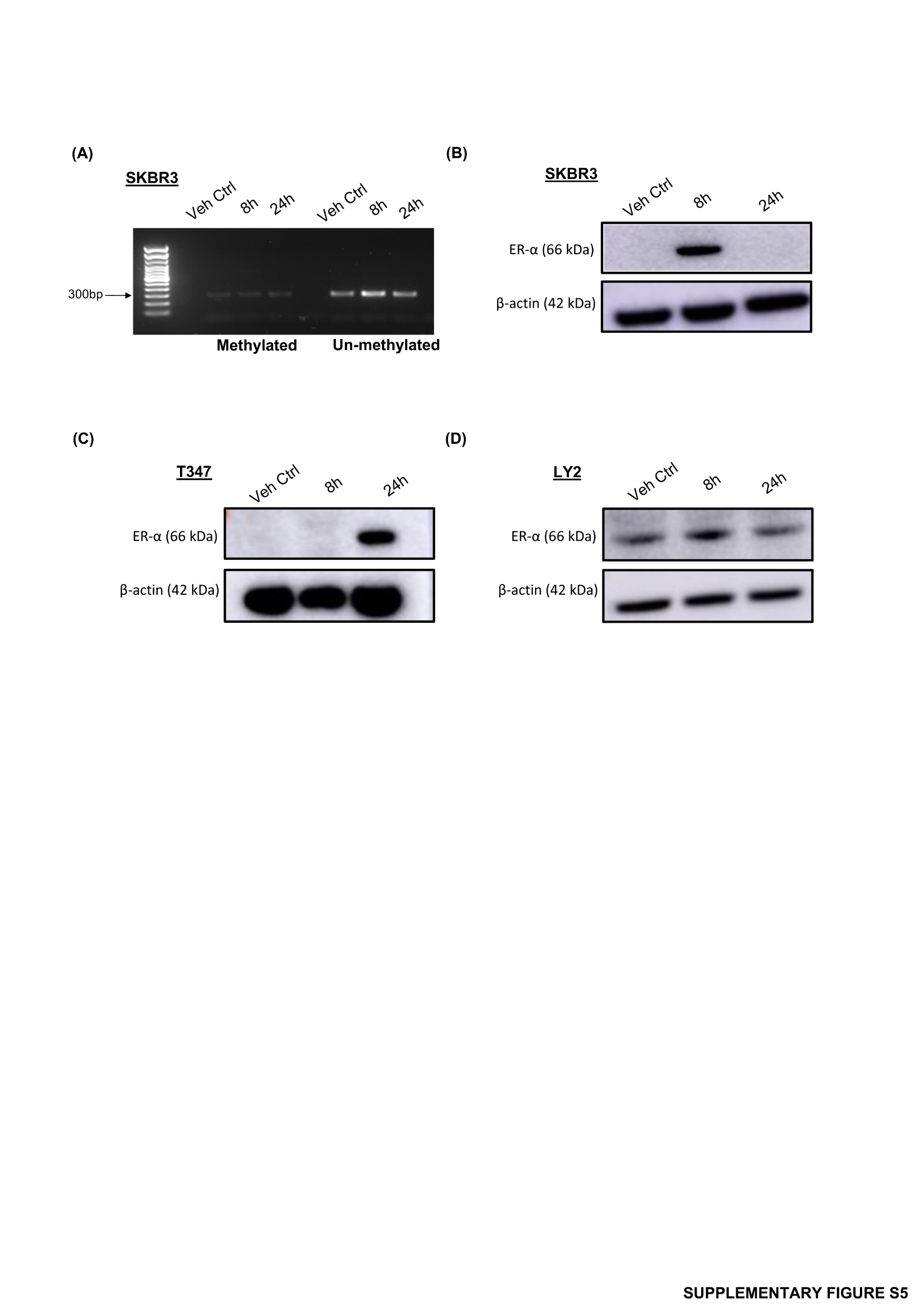


**Supplementary Figure S5. HER2-positive cell line validation of ESR1 methylation and ER expression changes.**

**(A)** DNA Methylation Analysis: Methylation-specific PCR (MSP) analysis was conducted on DNA from SKBR3 cells treated with 10 µg/mL trastuzumab or vehicle control for 8 and 24 hours. DNA was extracted, bisulfite-converted and amplified using methylation-specific primers. MSP products were visualized on a 2% agarose gel, differentiating between methylated and unmethylated DNA.

**(B, C, D)** Western Blot Analysis: SKBR3 (ER-negative, HER2-positive), T347 (ER-positive, HER2-positive), and LY2 (ER-positive, HER2-negative) cells were treated with 10 µg/mL trastuzumab or vehicle control for 8 and 24 hours. Total protein was extracted, quantified, and separated on 4–12% Bis-Tris gels. Membranes were probed for ER-alpha, with beta-actin used as a loading control. Bands were detected using enhanced chemiluminescence (ECL). ER-alpha expression was normalized to beta-actin and control samples. All experiments were performed in triplicate, and representative images are shown.

Abbreviations: MSP, methylation-specific PCR; HER2^+^, human epidermal growth factor receptor 2-positive; ECL, enhanced chemiluminescence.


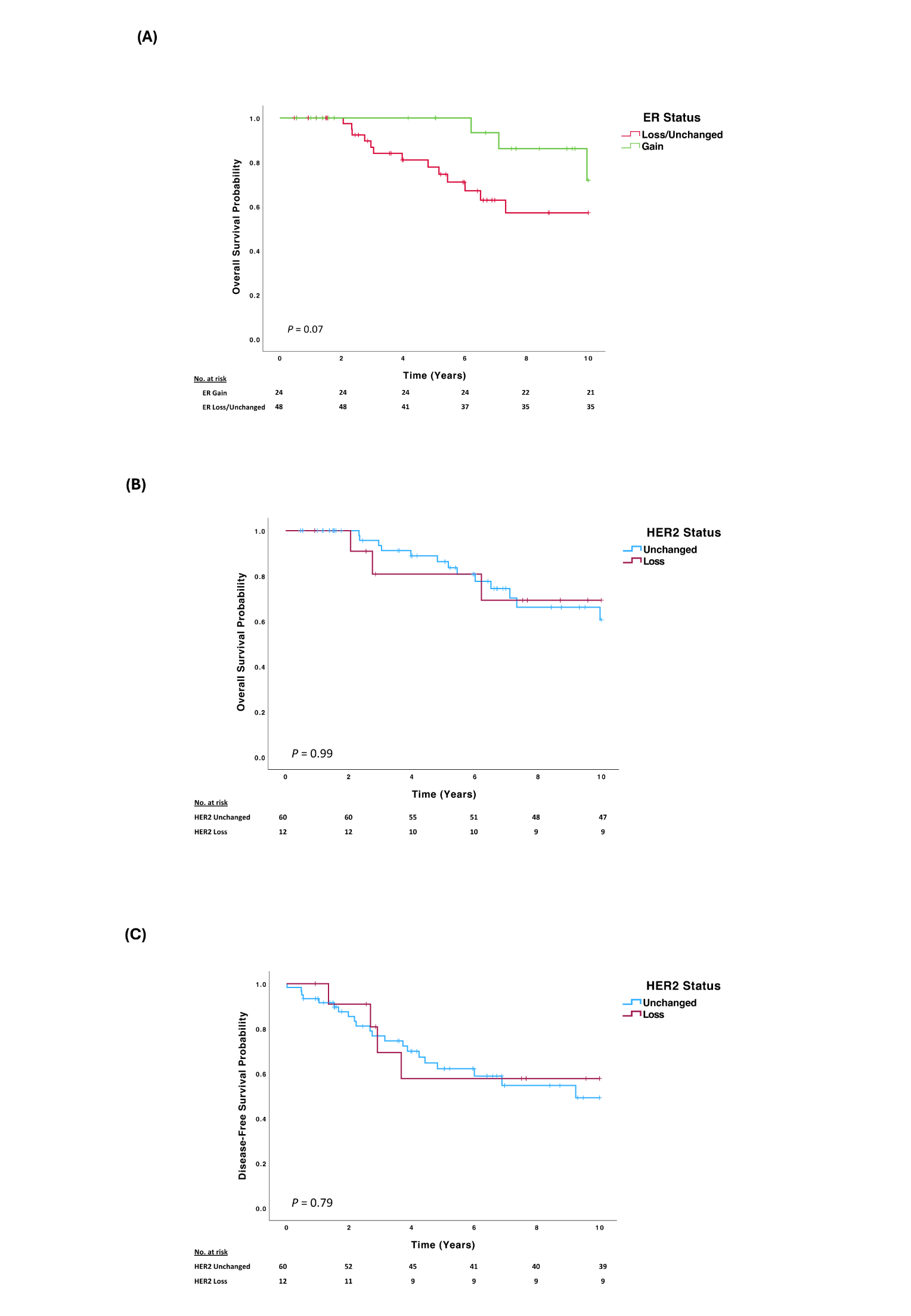


**Supplementary Figure S6. Clinical cohort survival analysis.**

**(A)** Kaplan-Meier plot of overall survival (OS) for patients who gained ER positivity versus those who had lost or unchanged ER positivity in post-treatment residual disease compared to pre-treatment biopsy (*P* = 0.07, log-rank).

**(B)** Kaplan-Meier plot of OS for patients with unchanged HER2 positivity compared to those who lost HER2 positivity in post-treatment residual disease compared to pre-treatment biopsy (*P* = 0.99, log-rank).

**(C)** Kaplan-Meier plot of disease-free survival (DFS) for patients who had unchanged HER2 positivity compared to those who lost HER2 positivity in post-treatment residual disease compared to pre-treatment biopsy (*P* = 0.79, log-rank).

Abbreviations: OS, overall survival; DFS, disease-free survival

**Supplementary Table S1. Summary of patient and tumor characteristics.**

[Excel]

**Supplementary Table S2. Differentially methylated CpGs in post-treatment vs. pre-treatment samples.**

[Excel]

**Supplementary Table S3. Methylation status of genes in top differentially methylated pathways in post-treatment vs. pre-treatment samples.**

[Excel]

**Supplementary Table S4. Differentially methylated CpGs in post-treatment samples with subsequent metastasis vs. pre-treatment samples.**

[Excel]

**Supplementary Table S5. Differentially methylated CpGs in metastatic vs. pre-treatment samples.**

[Excel]

**Supplementary Table S6.** **Methylation status of genes in top differentially methylated pathways in metastatic vs. pre-treatment.**

[Excel]

**Supplementary Table S7. Summary of ER and HER2 expression changes in clinical cohort.**

[Excel]
